# Impact of Enoxaparin on Maternal and Fetal Outcomes in In Vitro Fertilization (IVF) Pregnancies With Thyroid Autoimmunity: A Real-World, Single-Center, Retrospective, Observational Study

**DOI:** 10.7759/cureus.96633

**Published:** 2025-11-11

**Authors:** G. A. Rama Raju, Ponnada Sravani, Pranati Mahapatro, Aruna Kovvuri, Vishal Dave, Vivek Sharma, Deepak Bunger

**Affiliations:** 1 Department of Reproductive Medicine, Krishna IVF Clinic Private Limited, Visakhapatnam, IND; 2 Department of Medical Affairs, Intas Pharmaceuticals Limited, Ahmedabad, IND

**Keywords:** anti-thyroid antibodies (ata), enoxaparin, in-vitro fertilization (ivf), miscarriage prevention, observational study, pregnancy outcomes, preterm birth, retrospective studies, thyroid autoimmunity (tai)

## Abstract

Aim

Thyroid autoimmunity (TAI) in women undergoing in vitro fertilization (IVF) is linked to adverse pregnancy outcomes, despite euthyroid status. This study aims to evaluate the efficacy of enoxaparin in improving maternal and fetal outcomes in anti-thyroid antibody (ATA)-positive euthyroid IVF pregnancies.

Methods

This observational study included 80 ATA-positive women undergoing IVF. Levothyroxine was administered in hypothyroid women to achieve euthyroid status prior to embryo transfer, and enoxaparin therapy was initiated post-transfer and continued throughout pregnancy. The primary outcome was live birth rate. Secondary outcomes included obstetric complications - pregnancy-induced hypertension (PIH), gestational diabetes mellitus (GDM), intrauterine growth restriction (IUGR), miscarriage, and preterm delivery.

Results

The live birth rate was 78 (97.5%). Among single pregnancies, 44 (95.7%) reached full term, with only two (4.4%) resulting in preterm births. Twin pregnancies had a higher preterm birth rate of 11 (39.3%). The incidence of PIH was 11 (34.2%), with higher rates in twin pregnancies, seven (25.9%), compared to single pregnancies, four (8.3%). GDM was equally prevalent in both single and twin pregnancies (13 (26%) vs. 6 (21.4%)). A miscarriage was observed in two of the total cases. IUGR affected two (4.2%) of singleton and two (7.4%) of twin pregnancies. First-trimester bleeding occurred in five (10%) of singleton and three (10.7%) of twin pregnancies, while only one (3.6%) case of placental abruption was observed in twin pregnancies. No major safety concerns were observed.

Conclusion

Enoxaparin was associated with improved maternal and fetal outcomes, notably reducing the rates of miscarriage, preterm birth, and IUGR. These findings suggest that enoxaparin can be a valuable addition for improving pregnancy outcomes in ATA-positive patients undergoing IVF.

## Introduction

Thyroid autoimmunity (TAI) has been known to impact the maternal and fetal outcomes of pregnant women. Anti-thyroid antibodies (ATAs), including thyroperoxidase antibodies (TPOAbs), thyroglobulin antibodies (TgAbs), and thyroid-stimulating hormone (TSH) receptor antibodies (TRAbs), are markers of TAI or autoimmune thyroid disorder (AITD) [1]. The prevalence of TAI in women of reproductive age is estimated to be between 5% and 15%; the prevalence is even higher in pregnant women (5-20%) [2,3]. The presence of ATAs has been associated with adverse pregnancy outcomes such as miscarriage, preterm birth, intra-uterine growth restriction (IUGR), pre-eclampsia (PE), and pregnancy-induced hypertension (PIH) [4,5]. Traditionally, most of the available evidence linking TAI to adverse pregnancy outcomes has focused on women with overt thyroid dysfunction, particularly hypothyroidism [6], with clinical guidelines recommending levothyroxine supplementation as the primary treatment for improving pregnancy outcomes in this patient population [7]. However, approximately 75% of pregnant women with TAI exhibit euthyroidism [8]. Interestingly, studies have shown that the correction of thyroid hormone status in pregnant women associated with TAI was not adequate to prevent adverse maternal and fetal outcomes [2,6-9]. This highlights the need for a broader therapeutic approach in such patients.

The possible mechanisms behind the adverse pregnancy outcomes in ATA-positive pregnant women are mentioned below. TAI may cause an inflammatory state, characterized by an imbalance between pro-inflammatory T-helper (Th) 1 and Th17 cells and anti-inflammatory Th2 cells, regulated by T-regulatory (Treg) cells. Such immune-inflammatory dysregulation could disrupt implantation and fetal development, increasing the risk of pregnancy complications [10,11]. Hence, there are mechanisms independent of the thyroid hormonal status in ATA-positive pregnant women that need to be addressed.

Enoxaparin, a low-molecular-weight heparin, has gained attention in this context, mainly due to its dual anticoagulant and anti-inflammatory effects [12,13]. It has been used for women with thrombophilia or recurrent pregnancy loss (RPL), where it has been shown to improve live birth rates [14,15]. Beyond its anticoagulant properties, enoxaparin exhibits potential immune-modulatory effects, which may address both autoimmunity and inflammation [16,17], and could offer benefits in ATA-positive in vitro fertilization (IVF) patients, where immune-inflammatory dysregulation and hypercoagulability are more pronounced.

However, while meta-analysis and randomized controlled trials have explored the efficacy of enoxaparin in women with recurrent miscarriage and thrombophilia [14,15,18,19], its role in ATAs-positive IVF pregnancies (women undergoing IVF) is not well documented. Furthermore, there is a lack of clinical trials and real-world data from emerging economies, such as India, in this context. Hence, this real-world, single-center, retrospective, observational study was conducted to evaluate the role of enoxaparin in improving maternal and fetal outcomes in ATA-positive euthyroid women undergoing IVF.

## Materials and methods

Study design and settings

This was a single-center, investigator-initiated, retrospective, observational study conducted at Krishna IVF Clinic, Visakhapatnam, Andhra Pradesh, India, between January 2021 and May 2024. The medical records of pregnant women who conceived through IVF and tested positive for ATAs were reviewed for maternal and fetal outcomes. These women received injection enoxaparin sodium (injection Evaparin, manufactured by Intas Pharmaceuticals Ltd., Ahmedabad, Gujarat, India) 40 mg subcutaneously daily as part of their routine clinical care after embryo transfer (ET), continuing the treatment until the termination of pregnancy or successful delivery or baby birth. The study adhered to the guidelines of the Equator Network and followed the Strengthening the Reporting of Observational Studies in Epidemiology (STROBE) statement to ensure transparency and accuracy in reporting the results.

Study population

The study included infertile women between the ages of 20 and 40 years who tested positive for anti-thyroperoxidase (TPO) and/or anti-TgAbs and had undergone IVF. Eligible patients had a body mass index (BMI) of 18-40 kg/m^2^ and were diagnosed with infertility. Patients were excluded if they had a history of drug allergies, known infections such as HIV, hepatitis B, or hepatitis C, or any malignancy. Other exclusions included active major bleeding, thrombocytopenia, hyperandrogenism, hyperprolactinemia (serum prolactin level >30 ng/mL), and known or suspected endometriosis. Patients with uncontrolled diabetes, uncontrolled hypertension, liver or kidney disease, or suspected genital tract or breast cancer were also excluded.

Outcomes

The primary efficacy endpoint was the proportion of pregnancies resulting in live births. Secondary endpoints included the incidence of obstetric complications, such as PIH, oligohydramnios, IUGR, gestational diabetes mellitus (GDM), first-trimester bleeding and placental abruption, and fetal outcomes, including birth weight and proportion of preterm births. Incidence of adverse events (AEs) as documented in the treatment charts was recorded. Serious adverse events (SAEs) associated with enoxaparin, as well as any treatment discontinuations, were captured from the patient records.

Evaluation of pregnancy outcomes

Pregnancy outcomes were assessed using standardized definitions. GDM was diagnosed if fasting plasma glucose was ≥ 5.3 mmol/L (95 mg/dL), one-hour plasma glucose ≥ 10 mmol/L (180 mg/dL), or two-hour plasma glucose ≥ 8.6 mmol/L (155 mg/dL) [20]. PIH was defined as hypertension (blood pressure ≥140/90 mmHg) with or without proteinuria (≥300 mg/24 hours) emerging after 20 weeks of gestation, but resolving up to 12 weeks postpartum [21]. Placental abruption was classically defined as the complete or partial separation of a normally implanted placenta before delivery [22]. IUGR was defined as a disorder in which the fetus fails to reach its genetic development potential and is considered to be present when the weight at birth is less than the 10th percentile of the average for that gestational age [23]. Low birth weight was defined as fetal weight <2,500 g irrespective of gestational age [24]. Preterm birth was defined as any birth before 37 weeks of completed gestation [25]. Oligohydramnios was defined as decreased amniotic fluid volume (AFV) for that gestational age [26].

Statistical analysis

The sample size of 80 participants was determined based on the estimated incidence of adverse pregnancy outcomes among ATA-positive women undergoing IVF, as reported in previous literature. Prior studies have indicated a miscarriage rate of up to 25-30% in this high-risk population. Assuming an expected miscarriage rate reduction to 10% with enoxaparin intervention, a two-sided alpha level of 0.05, and a power of 80%, the minimum required sample size was calculated to be approximately 74 participants [14]. To account for potential dropouts and incomplete follow-up data, the final target sample was set at 80 patients. Demographic and baseline characteristics were analyzed using descriptive statistics. Categorical variables were expressed as frequencies and percentages, while continuous variables were summarized by count, mean, standard deviation, median, minimum-maximum values, and number of non-missing values, along with changes from baseline where appropriate. Chi-square test was used to find out any association between (1) obstetric complications in the present pregnancy with delivery outcomes in terms of singlet or twin delivery, as well as (2) delivery outcomes (singleton or twin delivery) and body weight at birth with time of delivery/gestational age (term or pre-term). All statistical analyses were carried out using SAS Version 9.3 (SAS Institute Inc., Cary, NC, USA). P-value < 0.05 was considered statistically significant.

## Results

Patients disposition and demographics

Data from 80 pregnant women with positive ATAs, conceived through IVF and treated with injection enoxaparin 40 mg, subcutaneously once daily, were analyzed. All patients were positive for at least one of the following ATAs - anti-TgAbs and anti-thyroid peroxidase (anti-TPO) antibodies at baseline. The majority of patients (n = 70, 87.5%) tested positive for anti-TgAbs, while 56 (70%) were positive for anti-TPO antibodies. The most common causes of infertility included polycystic ovarian disease (PCOD) (n = 15, 18.8%), adenomyosis (n = 12, 15%), and tubal factors (n = 8, 10%). Nineteen (23.8%) patients had a history of early pregnancy loss, and 10 (12.5%) and 13 (16.3%) patients had diabetes and hypertension, respectively. The baseline characteristics of these patients are summarized in Table 1.

**Table 1:** Patient Disposition and Baseline Characteristics * Data of 75/80 patients were available. TPO: thyroperoxidase; PCOD: polycystic ovary disease

Demographic Variables (N = 80)	
Age (years), mean ± SD (median, range)	30. 9 ± 3.6 (31, 17-40)
Married life (years), mean ± SD (median, range)	6.6 ± 2.3 (6.3, 2-12)
Body mass index (BMI), n (%)	
BMI < 30 Kg/m^2^	54 (67.5)
BMI ≥ 30 Kg/m^2^	26 (32.5)
Regularity of menstrual cycle, n (%)	
Menstrual cycle regular	51 (63.7)
Menstrual cycle irregular	29 (36.3)
Thyroid history, n (%)	
Euthyroid	30 (37.5)
Hypothyroid on levothyroxine	50 (62.5)
Patient with positive anti-thyroid antibodies, n (%)	
Anti-thyroglobulin antibodies positive	70 (87.5)
Anti-TPO antibodies positive	56 (70)
Anti-thyroid antibodies level (IU/mL), mean ± SD (median, range)	
Anti-thyroglobulin antibodies	205.5 ± 337.1 (70.3, 0-2373)
Anti-TPO antibodies	237 ± 321.2 (68.7, 0-1000)
Causes of infertility, n (%)	
PCOD	15 (18.8)
Endometriosis	4 (5)
Adenomyosis	12 (15)
Fibroid	7 (8.8)
Uterine anomaly	4 (5)
Tubal factor	8 (10)
Past history of pregnancy loss, n (%)	
Early pregnancy loss	19 (23.8)
Intra-uterine death/still birth	2 (2.5)
Second-trimester abortion	4 (5)
Past medical history, n (%)	
Diabetes	10 (12.5)
Hypertension	13 (16.3)
Past surgical history, n (%)	
Myomectomy	0
Cesarean section*	75 (100)
Polypectomy	8 (10)
Synechiae correction	2 (2.5)

Outcomes

A total of 78 (97.5%) of pregnant patients achieved live birth as a primary outcome. The secondary outcomes focused on assessing the incidence of various obstetric complications. The incidence of complications like PIH, oligohydramnios, IUGR, and first-trimester bleeding was lower, while GDM was higher in single versus twin pregnancies (Table 2). Chi-square test was used to find out any association between obstetric complications in the present pregnancy and delivery outcomes in terms of single or twin delivery. Only PIH was found to be significantly higher in the twin delivery group versus single delivery (25.9% vs. 8.3%, p < 0.05).

**Table 2:** Incidence of Obstetric Complications in Present Pregnancy * Single delivery (N = 50): n value for oligohydramnios, IUGR, PIH, GDM, and placental abruption was 48, 48, 48, 47, and 46, respectively. ** Twin delivery (N = 28): n value was 27 for each of IUGR, PIH, and GDM. Statistical test used: The Chi-square (χ^2^) test was applied to assess associations between obstetric complications and delivery type, as the data were categorical. P < 0.05 is considered statistically significant. ^†^ PIH was found to be significantly associated with delivery outcomes (P = 0.0489). IUGR: intrauterine growth restriction; PIH: pregnancy-induced hypertension; GDM: gestational diabetes mellitus

Obstetric Complications in Present Pregnancy	Single Delivery*, n (%) (N = 50)	Twin Delivery**, n (%) (N = 28)	χ^2^ Value	P-Value
Oligohydramnios	1 (2.1)	4 (14.3)	3.74	0.0531
IUGR	2 (4.2)	2 (7.4)	0.25	0.6153
PIH	4 (8.3)	7 (25.9)	3.88	0.0489^†^
GDM	13 (26)	6 (21.4)	0.07	0.7859
Bleeding in the first trimester	5 (10)	3 (10.7)	0.00	0.999
Placental abruption	0	1 (3.6)	0.83	0.3590

Among the delivery outcomes, 46 women had single deliveries, with 44 (95.7%) delivering at full-term and two (4.4%) delivering preterm. For twin deliveries, 17 (60.7%) were full-term deliveries, while 11 (39.3%) were preterm. Fetal weight outcomes revealed that 39 (83%) of single deliveries resulted in babies weighing ≥2.5 kg, and 43 (81.1%) of twin deliveries resulted in babies weighing less than 2.5 kg (Table 3). To evaluate any association between delivery outcomes (in terms of singleton or twin delivery) and time of delivery (term or pre-term), the Chi-square test was used. It was found that pre-term deliveries were significantly higher in patients who had twin delivery versus singleton delivery (39.3% vs. 4.4%, p = 0.0004). Also, it was found that there is an association between delivery outcome and low birth weight (<2.5 kg). It was found that the mothers having twin delivery had a significantly higher incidence of low birth weight babies than mothers who had singleton delivery (81.1% vs. 17%, p < 0.001).

**Table 3:** Delivery Outcomes and Baby Weight Distribution Statistical test used: The Chi-square (χ^2^) test was applied to determine the association between type of delivery (single vs. twin) and (a) gestational age at birth (preterm vs. term), and (b) neonatal birth weight (<2.5 kg vs. ≥2.5 kg). P < 0.05 is considered statistically significant. * Between delivery outcome and preterm/term delivery. There is a significant association between delivery outcome and term or preterm baby. The proportion of term babies is higher in both delivery outcomes. ** Between the delivery outcome and the weight of the baby. There is a significant association between delivery outcome and weight. The percentage of single deliveries is higher in >2.5 kg weight as compared to twin deliveries.

Delivery Outcomes	Preterm Delivery, n (%)	Term Delivery, n (%)	χ^2^ Value	P-Value
Single delivery (N = 46)	2 (4.4)	44 (95.7)	12.55	0.0004*
Twin delivery (N = 28)	11 (39.3)	17 (60.7)		
Baby weight	<2.5 kg, n (%)	≥2.5 kg, n (%)		
Single delivery (N = 47)	8 (17)	39 (83)	31.28	<0.001**
Twin delivery (N = 53)	43 (81.1)	10 (18.9)		

Safety

No SAEs were observed during the study. The treatment with enoxaparin was well-tolerated, with no documented cases of thrombocytopenia, thrombotic episodes, antepartum or postpartum bleeding, or allergic skin reactions. Additionally, there were no reports of injection site hematomas or significant subcutaneous bruising. No patients required discontinuation of enoxaparin due to adverse drug reactions. Overall, the safety profile of enoxaparin in this cohort was favorable, with no major safety concerns identified.

## Discussion

Infertility is defined as a disease characterized by the failure to establish a clinical pregnancy after 12 months of regular and unprotected sexual intercourse [27]. RPL, defined as the loss of two or more pregnancies [28], is a major challenge for physicians in the field of assisted reproductive technology (ART). Several disorders are known to contribute to RPL, including advanced maternal age, uterine malformations, genetic defects, endocrine disorders, infections, thrombophilia, and immunological disorders [29].

AITDs, caused due to immunological abnormalities, include Graves’ disease and Hashimoto’s thyroiditis as the most common AITDs [10]. Among the ATAs, TPOAbs, TRAbs, and TgAbs are directly correlated with AITD [30,31]. According to literature, there is a strong connection between adverse pregnancy outcomes and maternal thyroid disorders, including low fetal birth weight, anemia, hypertension, preterm labour, PE, IUGR, and stillbirth [32,33]. Relatively higher levels of TPOAbs have been reported in RPL patients versus normal women (5.4-20% vs 14-33%, respectively) [34]. Also, various studies have been conducted over time to evaluate the risk of infertility associated with TAI [35-42]. There is an average relative risk associated with TAI infertility. Several studies have highlighted the association between ATAs and miscarriages in women [39,43,44]. In these studies, a clear link between TAI, without overt thyroid dysfunction, is clearly associated with a significant increase in the miscarriage rate. These studies give credence to a hypothesis that TAI, independent of the thyroid hormone status, can adversely impact the maternal and fetal outcomes.

Among the probable mechanisms of ATAs affecting the maternal outcomes is an imbalance of T cells. In a normal pregnancy with healthy thyroid status, the frequency of Th2, Treg, and Breg cells and secretion of associated cytokines are increased. These cells are able to control the differentiation and function of inflammatory cells, as the frequency of Th1 and Th17 cells and their cytokines is downregulated. In case of AITDs such as Hashimoto’s thyroiditis, there is an increased Th1 and Th17 response along with their cytokines and a reduced Th2 and Treg cell frequency and function. Also, the natural killer cells (NK cells) and cytotoxicity are upregulated [10]. This creates a pro-inflammatory microenvironment and reproduction failure [45]. These activated inflammatory T cells in the uterus of ATA-positive pregnant women compromise the fetus, disturb the maternal tolerance, and may lead to adverse pregnancy outcomes [46].

Decidual NK (dNK) cells are the dominant cell type, forming 70% of the decidua's immune cell population in a normal pregnancy. These cells are required for trophoblast invasion, creating tolerance and maternal adaptation of the fetus [47]. Considering the high frequency and predominance of dNK cells, any disturbance in the number and function of these cells would compromise the pregnancy [48]. It has been confirmed that increased frequency and cytotoxicity of these immune cells are completely associated with adverse pregnancy outcomes [49-51]. There is evidence that B lymphocytes are involved in the pathogenesis of AITD [46]. These B cells lead to the production of non-organ-specific auto-antibodies that cross-react with trophoblast or placental antigens, activate complement, induce infiltration of inflammatory cells, cytokine production, and trigger a thrombotic cascade, which negatively affects both the mother and the fetus [52-54]. Abnormal cytokine production and imbalance (increased interferon γ (IFN-γ), decreased interleukin 4 (IL-4) and interleukin 10 (IL-10) levels) is associated with reproductive failure in women, as it may induce thrombotic events and inflammation in placental blood vessels. Elevated maternal TPOAbs hinder the thyroid gland’s response to human chorionic gonadotropin (hCG), which is crucial for fetal development, and these autoantibodies have been identified in neonates born to hypothyroid mothers [10].

Considering the hyper-inflammatory and hyper-thrombotic state produced due to ATAs present in pregnant women, there is a need to balance this state so as to prevent adverse pregnancy outcomes. Enoxaparin, with its anticoagulant and potential immune-inflammation modulatory properties [12], may offer a solution in ATAs-positive women undergoing IVF, but data on its effectiveness in this context remain limited. Herein, we provide real-world evidence on the use of enoxaparin in ATA-positive women undergoing IVF and its impact on maternal and fetal outcomes. Our findings suggest that dual anticoagulant and immuno-inflammatory modulation properties of enoxaparin may help mitigate the hyper-immune and hyper-thrombotic challenges faced by these patients.

In the present study, we evaluated the impact of giving enoxaparin to all ATA-positive pregnant women who underwent IVF-ET at Krishna IVF Clinic, Visakhapatnam, Andhra Pradesh, India. Enoxaparin at a dose of 40 mg subcutaneously per day was administered to all pregnant women after ET, till delivery or termination of pregnancy. On a similar note, Xu et al. [2] conducted a retrospective study (n=27,408) to evaluate the relationship between ATAs and adverse pregnancy outcomes in the Chinese population from 2013 to 2021. Another prospective study was conducted by Meena et al. [55] in the Indian population during 2013, where the impact of anti-TPO antibodies on pregnancy outcomes in euthyroid individuals was studied. In our study, the mean age (SD) of participants was 30.9 (3.6) years. This was comparable to that of Xu et al. [2] (32.1 years) and Meena et al. [55] (26.95 years). On comparing the mean BMI in our study (28.06 kg/m^2^) with Meena et al. [55] (22.47 kg/m^2^) and Xu et al. [2] (26.1 kg/m^2^), variations were noticed. These BMI differences may be attributed to social environment, geographical, lifestyle, or genetic variations, potentially influencing fertility outcomes and treatment response [56]. Higher BMI is often associated with poorer reproductive outcomes [57], which may partially explain some of the challenges faced by our cohort. The history of previous pregnancy loss in our study was higher than that reported by Bhattacharyya and co-workers [58] (25 (31.3%) vs. 6 (13%)) and Xu and colleagues [2] (25 (31.3%) vs. 850 (16%)), suggesting a higher risk population present in our cohort. This could indicate a more complex or severe reproductive history, necessitating more tailored clinical interventions. In terms of infertility causes, PCOD (15, 18.8%) was the most common factor in our study, aligning with its known prevalence among infertile women [59]. Other causes included adenomyosis (12, 15%), fibroid-related infertility (7, 8.8%), tubal factor (8, 10%), and endometriosis (4, 5%). The distribution of causes of infertility in our study is in line with global trends, though slightly higher for adenomyosis, indicating the potential impact of this condition on the reproductive health of our population [60,61]. Furthermore, a significant portion of patients had comorbid conditions, including hypertension (3, 16.3%) and diabetes (10, 12.5%), both of which are known to adversely affect pregnancy outcomes [62]. This higher prevalence of metabolic and cardiovascular conditions emphasizes the need for a comprehensive approach in managing infertility and pregnancy in this subgroup. These results highlight that while our population aligns with the broader literature on age and common causes of infertility, it also presents with higher BMIs and more complex medical histories, suggesting that patient management may need to be adjusted to account for these risk factors.

In our study, 50 (62.5%) patients were hypothyroid and were given levothyroxine therapy to achieve euthyroid status. This ensured that the impact of thyroid hormone status on pregnancy outcomes was taken care of. There have been several studies conducted to evaluate the impact of levothyroxine, independent of the thyroid hormone status, in patients with ATA-positive status; these studies concluded that levothyroxine does not improve pregnancy outcomes in euthyroid women having anti-TPO antibodies [9,63,64]. This raises important questions about the limitations of thyroid hormone replacement therapy in managing autoimmunity-related fertility issues. This highlights the need for alternative therapeutic strategies, such as enoxaparin, which may offer benefits beyond thyroid function normalization. Currently, there is a lack of studies on the potential therapeutic role of enoxaparin in ATAs-positive women undergoing IVF. Enoxaparin is known for its anti-inflammatory and anticoagulant properties [12,13] and might provide an adjunct benefit in managing the immune dysregulation associated with TAI. Our study provides some of the first real-world insights into this approach, suggesting that further investigation is warranted to evaluate the role of enoxaparin in this context.

The primary outcome of this study demonstrates a positive impact of enoxaparin on improving live birth rate. These results add to the growing body of evidence, offering new insights into the management of this high-risk population [14,15,65]. Our study observed a remarkably high live birth rate of 78 (97.5%) in women treated with enoxaparin. The secondary outcomes of this study reveal important insights into the management of obstetric complications in ATAs-positive pregnancies treated with enoxaparin, underscoring its potential role in mitigating risks associated with PIH, oligohydramnios, IUGR, first-trimester bleeding, placental abruption, and termination of pregnancy or miscarriage. The present study demonstrates that enoxaparin may have a positive impact on reducing the incidence of hypertensive disorders in pregnancy. While PIH occurred in a total of 11 (34.2%) of cases, this is slightly higher than the 12 (30%) incidence (one-third of those cases were PE and two-thirds were of gestational hypertension) reported by Meena et al. [55], suggesting a protective role for enoxaparin. The higher PIH rate in twin pregnancies (7, 25.9%) is expected and aligns with the literature that highlights an increased risk of hypertensive disorders in multiple gestations. Moreover, our results also support findings from Han and co-workers [66] that thyroid autoantibodies increase the risk of hypertensive disorders in euthyroid women, further suggesting that enoxaparin may help mitigate these risks, especially in ATA-positive pregnancies. Low molecular weight heparin (LMWH) has been reported to promote the transcription and release of placental growth factor (PlGF) from endothelial cells, providing a mechanistic basis by which LMWH could contribute to reducing the risk of PE [67].

The incidence of oligohydramnios was higher in twin pregnancies (4, 14.3%) than in singleton pregnancies (1, 2.08%), which is consistent with the increased risks associated with multiple gestations. Also, the rate of IUGR was notably low (2 (4.2%) in singleton and 2 (7.4%) in twin pregnancies), significantly lower than the rates reported by Meena et al. [55] (6, 15%) and Saki et al. 2014 [68] (21, 28%) in anti-TPO positive cases. This suggests that enoxaparin may improve placental function, reducing the occurrence of IUGR, possibly due to its immunomodulatory effects that improve placentation during implantation. The occurrence of PIH in the present study was four (8.3%) in singleton and seven (25.9%) in twin pregnancies, demonstrating a significantly higher risk in multiple gestations, which is well-documented in existing literature. However, the overall average incidence of PIH (14.7%) was lower compared with previous studies by Meena et al. [55] (30%) and Bhattacharyya et al. [58] (16.6%). This reduction may be attributed to early initiation of enoxaparin following ET, which likely improved placental perfusion and minimized endothelial dysfunction, thereby mitigating hypertensive complications. GDM was prevalent in 19 (24.4%) of cases, which is consistent with findings from studies of Tang et al. 2021 [69] and Ying et al. 2016 [70], where ATAs were associated with an increased risk of GDM. The use of assisted reproductive techniques and older maternal age may also contribute to this higher incidence, and while enoxaparin did not seem to reduce GDM, its role in improving other obstetric outcomes remains notable. Furthermore, the incidence of first-trimester bleeding and placental abruption was relatively low, with placental abruption observed in only 1.3% of enoxaparin-treated cases. LMWH prevents thrombo-inflammation in the placenta, thereby improving placentation [71] and thus may reduce the risk of abruption.

We also reported the incidence of pregnancy termination due to miscarriage, which was found to be remarkably low, with only 2.5% of cases experiencing miscarriage. This is significantly lower than the rates observed in various studies that have highlighted the increased risk of miscarriage associated with TAI. For example, a meta-analysis by Prummel and Wiersinga [72] calculated an odds ratio of 2.73 (95% confidence interval (CI) 2.20-3.40) for miscarriage in TAI-positive women, and reported a mean miscarriage rate of 27% (n = 298 of 1112), which is notably 10 times higher than the present study’s rate of 2.5%. Similarly, Busnelli et al. [73] in their systematic review and meta-analysis observed a significant increase in miscarriage with an odds ratio of 1.44 (95% CI (1.06-1.95); P = 0.02) among women with normal thyroid function but positive thyroid autoantibodies. These findings suggest that enoxaparin, used in this study immediately after ET, may play a role in reducing miscarriage rates by modulating the autoimmune response and improving pregnancy outcomes in ATA-positive women.

In the present study, the majority of pregnancies reached full term, with 44 (95.7%) of singleton pregnancies and 17 (60.7%) of twin pregnancies resulting in in-term deliveries (singlet vs. twin pregnancy, p < 0.0004). The slightly elevated rate of preterm births (n = 11, 39.3%) in twin pregnancies is consistent with the known risks associated with multiple gestations and IVF treatments. This is an expected finding, given the higher likelihood of complications in twin pregnancies, which often include preterm delivery. In singleton pregnancies, our study observed a notably low preterm delivery rate of 4.4% (2/46), which is significantly lower than those reported in other studies, such as 14.3% in Bhattacharyya et al. [58] 12.5% in Meena et al. [55] and 8.95% in Xu et al. [2] The lower preterm delivery rate in our study may be attributable to the early initiation of enoxaparin immediately after ET and its continuation until delivery, suggesting that enoxaparin may improve placentation there by decreasing the chances of preterm delivery. In comparison, the study by Saki and co-authors [68] reported a much higher incidence of preterm delivery at 48.1% in anti-TPO-positive patients, further emphasizing the potential benefit of enoxaparin in reducing preterm risks. Moreover, our findings are consistent with the meta-analysis by Thangaratinam et al. [74], which highlighted the association between maternal thyroid autoantibodies and adverse pregnancy outcomes, such as miscarriage and preterm delivery. Our results indicate that the administration of enoxaparin may mitigate these risks, as evidenced by the much lower incidence of preterm births in our cohort, especially in singleton pregnancies.

The majority of newborns from singleton deliveries had a birth weight of ≥2.5 kg, indicating healthy fetal growth. In contrast, twin pregnancies were associated with a higher incidence of low birth weight, a common outcome in multiple gestations due to restricted intrauterine space and higher rates of preterm delivery (singlet vs. twin deliveries, p < 0.0001). Singleton pregnancies were predominantly full-term with normal birth weight infants, reflecting favorable outcomes possibly enhanced by enoxaparin’s role in improving placental perfusion and reducing thrombo-inflammatory events. Chen et al. [75] showed an association between TPOAbs positivity and low birth weight. Our study demonstrates that despite the presence of ATAs, enoxaparin treatment may support favorable birth outcomes, particularly in singleton pregnancies. Additionally, there was no perinatal mortality in our study. Another observation study conducted by Männistö et al. in 2009, where the perinatal outcomes were evaluated in ATA-positive pregnant women, it was found that the perinatal mortality rate was high (2.4%) in anti-TPO antibody-positive pregnant women [76]. The absence of perinatal mortality in our study could be attributed to the protective effects of enoxaparin, further underscoring its potential benefit in ATA-positive IVF pregnancies. No serious adverse effects were noticed during the study. This is consistent with previous literature showing that enoxaparin is generally well tolerated in pregnant women [15,65,77]. The absence of SAEs in our study reaffirms the safety of enoxaparin in women undergoing IVF, especially when administered at a prophylactic dose of 40 mg/day. Notably, none of the participants required discontinuation of enoxaparin due to adverse effects, further highlighting its tolerability in this patient population.

Among the major limitations of our study are the retrospective nature, small sample size, a lack of a comparator group, the presence of other causes of infertility, and adverse maternal outcomes such as pre-existing diabetes and hypertension. A large randomized clinical trial with a long-term follow-up would be required to further confirm the outcomes observed in our real-world study.

## Conclusions

In a nutshell, this study highlights the potential therapeutic benefits of enoxaparin in improving maternal and fetal outcomes in ATA-positive IVF pregnancies. The remarkably low rates of miscarriage, preterm delivery, and IUGR observed in this cohort suggest that enoxaparin may contribute to better placental function and reduced obstetric complications. Additionally, the high live birth rates and low incidence of complications further underscore the potential of enoxaparin as a valuable adjunct in managing high-risk pregnancies associated with TAI.
